# Evidence of co-infection of chikungunya and densonucleosis viruses in C6/36 cell lines and laboratory infected *Aedes aegypti *(L.) mosquitoes

**DOI:** 10.1186/1756-3305-3-95

**Published:** 2010-10-12

**Authors:** Aruna Sivaram, Pradip V Barde, Mangesh D Gokhale, Dinesh K Singh, Devendra T Mourya

**Affiliations:** 1 Microbial Containment Complex, National Institute of Virology, Sus Road, Pashan, Pune 411 021, India

## Abstract

**Background:**

Densonucleosis viruses are the etiological agents of insect's disease. We have reported the isolation of densovirus from India and its distribution among the natural populations of *Aedes aegypti *mosquitoes across the country. Since densonucleosis virus persistently infects mosquito populations, and is demonstrated to negatively affect multiplication of dengue virus in *Aedes albopictus*, it would be interesting to study if this virus has a role in determining the susceptibility of the vector mosquito *Ae. aegypti *to chikugunya virus.

**Methods:**

Mosquito cell lines and adult *Ae. aegypti *mosquitoes infected with densovirus were superinfected with Chikungunya virus and both the viruses were quantitated by determining their genomic copy number by real time amplification. Comparison was made between the log of genomic copy numbers of the viruses in the presence and absence of each other.

**Results:**

The log of copy number of the viruses did not vary due to co-infection. Even though the RNA copy number of chikungunya virus increased over the period of time, no change was observed in the RNA copy number between the control and the co-infected group on any given day. Similarly, DNA copy number of densovirus also remained unchanged between the control and the co-infected groups.

**Conclusion:**

Chikungunya virus neither stimulates the replication of densovirus nor is its own replication suppressed due to co-infection. *Ae. aegypti *mosquitoes with densovirus infection were as susceptible to infection by chikungunya virus as the uninfected mosquitoes.

## Background

Densonucleosis viruses (DNV) are known to persistently infect the mosquito cell lines and mosquito populations in nature. They belong to the family Parvoviridae and genus Brevidensovirus. DNV have been isolated from several species of mosquitoes like *Culex salinarius *[1], *Aedes aegypti *[2,3], *Ae. albopictus *[4], *Anopheles minimus *[5] and mosquito cell lines [6]. They are isometric, non-enveloped viruses of about 20 nm in diameter with single stranded linear DNA, packaged either as plus or a minus strand [7]. Palindromic sequences that form hair pin structure are found in both the ends of the genome [8,9]. The coding sequences include the genes for non structural proteins NS1 and NS2 occupying the 5' end of the strand and the genes for structural protein VP occupying the 3' end [3,9,10].

DNV is pathogenic to mosquitoes, resulting in loss of mobility, deformity and loss of pigmentation in the mosquito larvae. A very high rate of mortality (up to 90%) was reported when first instar *Ae. aegypti *larvae were exposed to *Ae. albopictus *DNV [11]. The pathogenicity of the virus is dose dependent, where in at lower doses of infection with DNV, the larvae survive to form adults that carry the infection [4,12]. Virus multiplication has also been observed in the ovaries of the infected mosquitoes, which enable them to transmit the virus vertically to the next generation [11,13]. DNV has a role in significantly reducing the vectorial capacity of the mosquitoes by reducing mosquito lifespan [13]. DNV has also been developed as a transducing vector used to introduce constructs that interfere with the arboviral infection in mosquitoes.

Vector competence refers to the intrinsic permissiveness of an arthropod vector for infection, replication and transmission of a vertebrate pathogen [14]. Vector competence is governed by intrinsic (genetic) factors and extrinsic factors [15,16]. The microflora present in the midgut of mosquito might also have a role in determining their susceptibility to the viruses [17]. Since DNV has been seen to be persistently infecting mosquito populations, it would be interesting to study if this virus has a role in determining the susceptibility of the vector to other arboviruses. C6/36 mosquito cell cultures persistently infected with AalDNV showed markedly lower CPE than naive-cell cultures or acutely AalDNV infected cultures when super-challenged with DENV-2 [18]. Paterson *et al *[19] reported that persistent infection with the low virulence type of DNV did not prevent severe CPE due to super-challenge with a more virulent type. DNV infected *Ae. albopictus *mosquitoes have been reported to show reduced numbers of DENV-2 when super challenged as compared to those uninfected with DNV [20]. Their study also showed that super-challenge with DENV-2 stimulated DNV replication. Recent studies show that mosquito cells can accommodate balanced, persistent co-infections with a DNV and DENV [21]. When cells dually infected with DENV and DNV were super-challenged with Japanese encephalitis (JE), cultures were stable without signs of cytopathology, with 99% cells producing antigens of all the three viruses [22].

Chikungunya fever (CHIK) is a viral disease transmitted by *Aedes *mosquitoes. CHIKV belongs to the family Togaviridae, genus *Alphavirus*. The disease has a significant potential to spread globally given the wide distribution of its arthropod vector [23,24]. In India CHIKV re-emerged after nearly 32 years in October 2005 in a very high magnitude. During this outbreak of CHIKV, our laboratory received mosquitoes from which we had isolated and characterized *Ae. aegypti *DNV and studied its distribution among different *Ae. aegypti *populations across India [3]. Whether the susceptibility of *Ae. aegypti *mosquitoes to CHIKV is affected due to coinfection of CHIKV and DNV is unknown. The present communication focuses on the effect of co-infection of DNV and CHIKV in the mosquito cell line and in *Ae. aegypti *mosquitoes.

## Methods

### Virus stocks

Approximately 10,000 fourth instar larvae from the DNV infected colony of *Ae. aegypti *maintained at National Institute of Virology (NIV), Pune were triturated in 150 ml of Phosphate Buffer Saline (PBS) using a tissue homogenizer (Fisher Scientific). The resulting slurry was centrifuged at 10,000 rpm for 30 min at 4°C to remove the cell debris. The supernatant was aliquoted and stored at -80°C until used. CHIKV isolate (Strain No: 601573) was obtained from virus repository of this institute and stock was prepared in *Vero E6 *cell lines. These viral stocks were used throughout the study. Both the virus stocks were quantitated by real time PCR.

### Cell lines

C6/36 cells were grown in Mitsuhashi Maramorosch (MM) media [25], supplemented with 10% fetal bovine serum (FBS) and 1% penicillin - streptomycin. The cell line was maintained at 28°C.

### Mosquitoes

The *Ae. aegypti *mosquitoes used for these studies were from an insectary maintained at NIV, Pune for the last 35 years. The mosquitoes were maintained at 28 ± 2°C and a relative humidity of 70-80%. The larvae were fed on sterilized yeast tablets and dog biscuits mixed in the proportion of 70 and 30%, respectively. The adults were reared in cages and were fed on 10% glucose. Female mosquitoes were fed with chicken blood every third day to obtain eggs.

### Primer designing for SYBR GREEN real time PCR

Primers for real time PCR of DNV was designed using sequences of *Ae. aegypti *DNV (Accession no: FJ360744), which was isolated from India [3]. For CHIKV, primers were designed from envelope gene (E1) using sequences of Indian strains of CHIKV available in the NCBI genebank. The software Primer Express 3.0 (Applied Biosystems) was used for the primer designing. For preparation of standards for quantitation of CHIKV, the T7 polymerase site was added to the primer which was previously described by Hasabe *et al *[26]. The details of primers are given in table 1.

**Table 1:** Primers designed for real time PCR for DNV and CHIKV

Virus	Name of Primer	Sequences	Genomic Location
DNV	Denso SYBR Fwd	CAACGCTTGCTAACGGGAACGAC	2975-2997
	
	Denso SYBR Rev	CAGTTGCTGCTGCTGATGTTAATCCGA	3072-3098

CHIKV	CHIK Q Fwd	TGGAGAAGTCCGAATCATGC	10316-10335
	
	CHIK Q Rev	TAACTGTGACGGCATGGT	10445-10462

*The sequence of the T7 polymerase site is represented in italics and underlined.

### SYBR Green I quantitative real time PCR of DNV

DNA extracted from DNV stock using QiaAmp DNA Mini kit (Qiagen, Hilden, Germany) was amplified with DNV specific primers [20]. The amplicon of 1.1 kb was cloned into *E. coli *cells using TA cloning kit (Invitrogen, Carlsbad, CA) following manufacturer's protocol. Blue white colony screening was used to select the transformed cells. Plasmids were extracted from positive clones using Plasmid Mini kit (Qiagen, Hilden, Germany) and the insert was sequenced on an ABI 3100 automated DNA sequencer using Big Dye terminator kit (Applied Biosystems). The concentration of the plasmid was determined using NanoDrop spectrophotometer (NanoDrop technologies, Inc., USA). The plasmid was serially diluted 10 fold and used as standard for real time PCR. The reaction mixture for real time amplification composed of 12.5 μl of *Power *SYBR Green PCR Master Mix (Applied Biosystems, Foster City, CA), 1 μl of each primer (10 pico moles) and 5 μl of template DNA. The mixture was made up to 25 μl by adding 5.5 μl of water. The real time PCR conditions for DNV were 10 min of initial hot start at 95°C, denaturation at 95°C for 15 sec and 1 min of annealing and extension at 60°C for 40 cycles. The reactions were carried out in ABI 7300 real time PCR system (Applied Bio-systems). The system software provided (SDS) was used to analyze the results by plotting standard curve.

### SYBR Green I quantitative real time RT-PCR of CHIKV

RNA was extracted from CHIKV stock using QIAamp viral RNA mini kit (Qiagen, Hilden, Germany) and was reverse transcribed to cDNA using Reverse transcription system (Promega Corp., Madison, USA) according to the manufacturer's protocol. The reverse primer designed for real time amplification of CHIKV was used to synthesize cDNA. The forward primer used to amplify the cDNA contained a site of T7 polymerase which could be anchored to the amplicon to enable *in vitro *transcription. The amplicon of 250 bp was subjected to *in vitro *transcription using T7 RNA polymerase by Riboprobe *in vitro *transcription system (Promega Corp., Madison, USA) according to manufacturer's instructions. The transcript was quantitated using NanoDrop spectrophotometer. The RNA was reverse transcribed to cDNA and serially diluted 10 fold, which served as standard. The reaction mix for real time amplification was prepared similar to that of DNV. The real time amplification conditions were 10 min of initial hot start at 95°C, denaturation at 95°C for 15 sec and 30 secs of annealing at 50°C and 30 secs of extension at 62°C for 40 cycles. Results were analyzed by plotting standard curve using SDS software.

### Determining the effect of co-infection of CHIKV and DNV in C6/36 cell line

C6/36 cells were counted using hemocytometer and were seeded in 6 well plates at 3000 cells per well. When the cells were 80% confluent, the media was removed and the cells were infected by adding DNV to the cell sheet. The quantity of DNV used to infect corresponded to 6.07 × 10^8 ^viral particles. The cells were incubated at 28°C for one hour with intermittent rocking of the plate at every 15 min to enable adsorption of the virus. After incubation, the inoculum was removed, and the cells were washed with sterile PBS, fed with 2 ml of fresh media supplemented with 2% FBS and returned to the incubator.

The cells infected with DNV were incubated for two days. On day 3 post DNV infection, the cells were superinfected with 5.73 × 10^5 ^RNA copy number of CHIKV. All infections and superinfections were done in triplicates. Appropriate controls were used which include cells infected with only CHIKV, only DNV and uninfected cells.

Virus was harvested from the infected cells every 24 hr post superinfection for 4 consecutive days by repeated freeze thaw cycles and centrifugation at 5000 rpm for 20 mins at 4°C. The supernatant was divided into two batches, one of which was used to quantitate DNV and the other to quantitate CHIKV. Nucleic acids extracted from these samples were amplified real time to quantify the viruses using specific primers. Standards were also amplified along with the samples and the viral genomic copy numbers were quantitated' by standard curve analysis. Each sample was amplified in triplicates by real time PCR. The copy number of genomes of DNV and CHIKV in the presence and absence of each other was compared.

### Determining the effect of co-infection of CHIKV and DNV in *Ae. aegypti* mosquitoes

First instar *Ae. aegypti *larvae after one hour of hatching, (n = 3000) were counted and transferred to a beaker containing 180 ml of water and infected with DNV by adding 20 ml of virus stock to the water in which the larvae were reared. The quantity of DNV used for infection corresponded to 6.07 × 10^8 ^viral particles. After 48 hr, the larvae were transferred to a larger pan, fed on a mixture of yeast tablet and dog biscuits and were maintained under normal insectary conditions. Many larvae died during the course of development. Among the females that emerged, 5 individuals were randomly checked by PCR for DNV infection. The remaining females were counted and were infected orally by membrane feeding on heparinised blood containing 5.73 × 10^5 ^RNA copy number of CHIKV (5 IU/ml of blood-virus mixture) as described by Harada *et al *[27]. The mosquitoes were allowed to feed for 45 minutes and the fully engorged mosquitoes were segregated and counted. They were maintained under normal insectary conditions with 10% glucose as a nutritional supplement. Appropriate controls were used which include mosquitoes infected with only CHIKV, only DNV and uninfected mosquitoes.

Twelve mosquitoes were collected and stored in 2 batches of 6 mosquitoes each on alternate day post infection starting from day 0 up to day 8. DNA was extracted from one batch to analyse for DNV and RNA from the other batch to analyse for CHIKV.

Nucleic acid extraction was done from individual mosquitoes by triturating the whole mosquito in Nuclease free water. The nucleic acids were then quantitated by real time amplification along with the standards using the corresponding primers and analyzed by plotting standard curve. The real time amplification of each sample was performed in triplicates.

## Results

### SYBR Green I quantitative real time PCR of DNV

**Figure 1:**
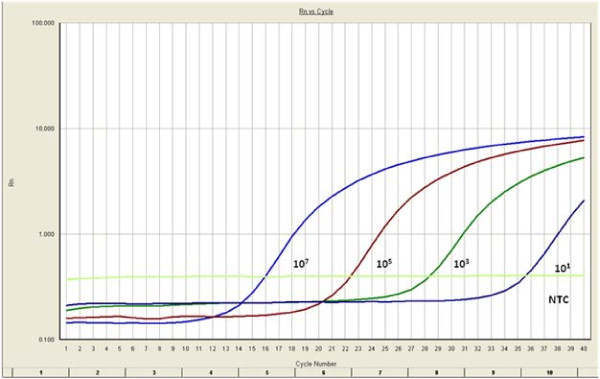
**Amplification plot generated for real time amplification of DNV**. Plasmid standards were prepared, serially diluted and amplified real time using primers for DNV. The real time PCR was sensitive enough to detect 10 copies of the standard.

**Figure 2:**
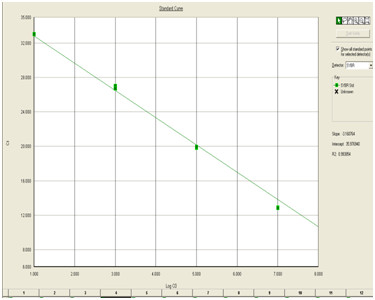
**Standard curve generated for real time amplification of DNV**. Plasmid standards were diluted serially and amplified along with samples. Quantitation was done by plotting the standard curve using SDS software.

The amplification plot (Fig 1) gave sigmoid curve and the melting curve analysis showed a single dissociation peak with a *Tm *of 77.3°C, indicating absence of primer dimer. Standard curve analysis (Fig 2) showed R^2 ^value of 0.99 and slope of -3.16. The real time assay for DNV was sensitive enough to detect 10 copies of the standard.

### SYBR Green I quantitative real time PCR of CHIKV

**Figure 3:**
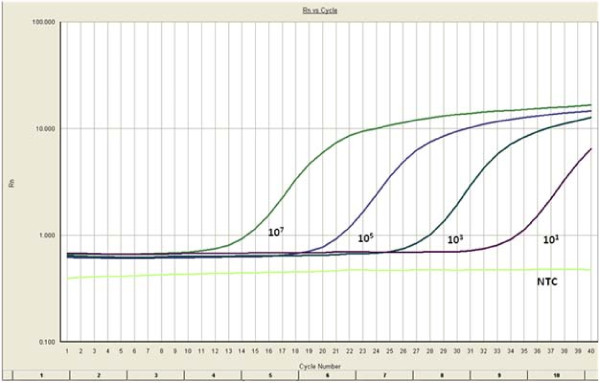
**Amplification plot generated for real time amplification of CHIKV**. RNA standards were prepared, serially diluted and amplified real time using primers for CHIKV. The real time PCR was sensitive enough to detect 10 copies of the standard.

**Figure 4:**
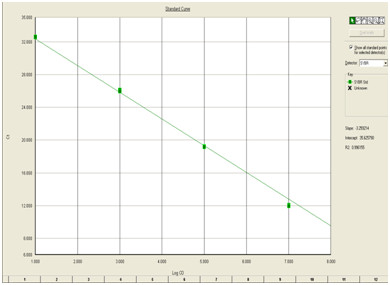
**Standard curve generated for real time amplification of CHIKV**. RNA standards were diluted serially and amplified along with samples. Quantitation was done by plotting the standard curve using SDS software.

Melting curve analysis showed a single dissociation peak with a *Tm *of 80.1°C. The amplification plot analysis (Fig 3) gave a sigmoid curve. Standard curve analysis (Fig 4) showed a R^2 ^value of 0.99 and slope of -3.25. A lowest copy number of 10 copies could be detected in the standard.

### Determining the effect of co-infection in mosquito C6/36 cells

When nucleic acids were quantified by real time PCR, it was found that the copy number of the genome of neither CHIKV nor the DNV was affected due to co-infection (*p *> 0.01). On day 1 post infection, log of RNA copy number of CHIKV was found to be 10.330(± 0.004) in the presence of DNV and 10.051(± 0.006) in the absence of DNV. Though there was a progressive increase in the copy number of CHIKV on each day post infection, no change was observed between the DNV infected and uninfected samples. There was also no change in the copy number of DNV due to infection with CHIKV. The log values of genomic copy number are given in Table 2.

**Table 2:** Effect of co-infection of DNV and CHIKV in C6/36 cell lines. The values are approximated to three decimal points.

Days post infection	LOG of RNA copy number of CHIKV (± SD)		LOG of DNA copy number of DNV (± SD)	
	
	In presence of DNV	In absence of DNV	In presence of CHIKV	In absence of CHIKV
1	10.330(± 0.004)	10.051(± 0.004)	6.227(± 0.044)	6.040(± 0.034)

2	11.442(± 0.013)	10.643(± 0.017)	6.472(± 0.003)	5.693(± 0.048)

3	11.664(± 0.132)	11.184(± 0.041)	6.267(± 0.020)	5.861(± 0.063)

4	11.785(± 0.024)	11.200(± 0.009)	6.025(± 0.102)	5.881(± 0.006)

### Determining the effect of co-infection in *Ae. aegypti* mosquitoes

Mosquito samples were subjected to real time PCR using specific primers for DNV and CHIKV. It was found that there was no change in the copy number of the genome of either CHIKV or the DNV due to co-infection (*p *> 0.01). The RNA copy number of CHIKV was found to be 3.345(± 0.130) on day 0 post infection in the presence of DNV, while it was 3.173(± 0.081) in the absence of DNV. On day 8, the log of CHIKV copy number increased to 5.354(± 0.110) in the presence of DNV and 5.269(± 0.237) in the absence of DNV. Though there was an increase in RNA copy number of CHIKV by 2 logs between day 0 and day 8, no change was observed between DNV infected and uninfected mosquitoes on any day post infection. Similarly, log of DNA copy number of DNV also did not show any variation due to the presence or absence of CHIKV. The values are represented in table 3.

**Table 3:** Effect of co-infection of DNV and CHIKV in *Ae. aegypti *mosquitoes. The values are approximated to three decimal points.

Days post infection	LOG of RNA copy number of CHIKV (± SD)		LOG of DNA copy number of DNV (± SD)	
	
	In presence of DNV	In absence of DNV	In presence of CHIKV	In absence of CHIKV
0	3.345(± 0.130)	3.173(± 0.081)	1.906(± 0.185)	2.389(± 0.308)

2	3.220(± 0.501)	3.573(± 0.554)	1.709(± 0.205)	2.642(± 0.029)

4	3.613(± 0.482)	3.807(± 0.057)	1.040(± 0.338)	1.574(± 0.308)

6	3.893(± 0.542)	3.897(± 0.601)	1.409(± 0.307)	1.043(± 0.105)

8	5.354(± 0.110)	5.269(± 0.237)	0.765(± 0.050)	1.410(± 0.364)

## Discussion

DNV are among the most important mosquito pathogenic viruses found in natural mosquito populations. Studies at the laboratory level show them to be highly detrimental to the mosquito populations when early instar larvae are infected. The virus has also been used as a transducing agent to deliver genes of interest into the mosquitoes. Infection with DNV reduces the life span of mosquitoes there by altering its vectorial capacity. There were also a few reports about the virus altering the vector competence of mosquitoes. When AalDNV infected cultures or *Ae.albopictus *mosquitoes were super-challenged with DENV-2, the cell lines showed lower CPE and the mosquitoes showed lesser number of DENV [18,20]. Later studies showed that DENV and DNV can stably co-exist in cell lines. When these dually infected cells were super challenged with JEV, no signs of cytopathology were observed [21,22]. Given the re-emergence of CHIKV and its potential to spread far and wide, it would be of significance to study whether infection with DNV alters the susceptibility of *Ae. aegypti *to CHIKV.

The mosquito cell lines and mosquitoes were first infected with a known quantity of DNV. This was followed by incubation for a certain interval of time so that DNV could establish itself in the system. Then the cells and individual mosquitoes were infected with CHIKV. Throughout this study we have quantitated the genomic copy number of the viruses by real time amplification, which in turn implies to the quantity of the virus in each sample. Direct methods of quantitation like plaque assays and TCID_50 _are not feasible for DNV [28] since they do not produce morphological changes in cells. For appropriate comparison, it is required that CHIKV is also quantitated through the same means.

This study shows that CHIKV neither triggers the replication of DNV nor is its own replication suppressed due to co-infection. *Ae. aegypti *mosquitoes with DNV infection were found to be as susceptible to CHIKV infection as uninfected or lowly infected natural population of mosquitoes [3]. There was an increase in the copy number of CHIKV on each day post infection as expected. But the copy number of CHIKV remained more or less constant between the DNV infected and uninfected group on any given day i.e. the multiplication of CHIKV was not affected by presence of DNV in the same system. There was no increase in the levels of DNV over time. This could be because DNV attains maximum growth on day 3 post infection [28]. In our study, we have done superinfection on day 3 post DNV infection. By this time DNV would have attained maximum growth and hence the level of DNA copy number remained more or less constant on the following days (table 2).

The difference of around 0.1-0.8 log in the genomic copy number of the viruses in the presence and absence of co-infection could be attributed to the individual mosquito rather than any real change in the virus particles. This change might not reflect the actual number of infectious particles. Statistical analysis of the data by t-test also suggests that the genomic copy number of neither of the viruses was altered significantly due to co-infection *in vitro *and *in vivo *(p > 0.01).

A phenomenon called viral accommodation has been reported in shrimp where in natural multiple infections are possible [29,30]. We observed a similar phenomena here when mosquitoes were co-infected. The viruses could stably co-exist both in the cell lines and adult mosquitoes. This is further ascertained by the fact that DNV was found infecting natural populations of *Ae. aegypti *across India including states like Kerala, Andhra Pardesh, Karnataka and Gujarat [3], which faced severe CHIKV outbreak. The vector mosquitoes continued to transmit the arbovirus despite DNV infection. DNV being a DNA virus has its replication in nucleus whereas CHIKV being a RNA virus replicates in cytoplasm and probably because of this, their replication might not interfere with that of each other.

This study shows that neither CHIKV nor DNV influence the multiplication of each other in mosquito cell lines or mosquitoes.

## Competing interests

The authors declare that they have no competing interests.

## Authors' contributions

AS and PVB performed real time PCR and cell culture experiments. MDG and DKS were involved in mosquito experiments. DTM, AS and PVB were involved in designing the experiments and preparing the manuscript. All authors read and approved the final manuscript.
